# Two is more valid than one, but is six even better? The factor structure of the Self-Compassion Scale (SCS)

**DOI:** 10.1371/journal.pone.0207706

**Published:** 2018-12-05

**Authors:** Sonja Kumlander, Oskari Lahtinen, Tiina Turunen, Christina Salmivalli

**Affiliations:** 1 Department of Psychology, University of Turku, Turku, Finland; 2 Shandong University, Shandong, China; Universiti Sains Malaysia, MALAYSIA

## Abstract

**Introduction:**

Self-compassion refers to a non-evaluative, interconnected and mindful attitude towards oneself especially when facing difficulties or feelings of personal inadequacies. The Self-Compassion Scale (SCS) is a frequently used instrument designed to measure self-compassion either by using the six subscale scores, or by calculating a total score, averaged across all 26 items.

**Purpose:**

The purpose of this study is to examine the factor structure of the Self-Compassion Scale, and in particular, whether the widely used six-factor model and the unidimensional model can be confirmed.

**Methods:**

The internal structure of the SCS was examined using confirmatory factor analysis (CFA). Six different models (a one-factor model, an oblique six-factor model, a higher-order model, an oblique two-factor model, a bi-factor model with one general factor (bifactor model) and a bi-factor model with two general factors, i.e. two-bifactor model) were tested in a sample of adolescents (n = 1725; 50.3% female; mean age = 16.56, SD = 1.95). All models were replicated using responses collected five months after the first data collection from 1497 students (W2), who were largely, but not completely, the same students involved in W1 data collection.

**Results:**

Fit indices for the two-factor model implied an acceptable fit, but none of the remaining models tested met the criteria for an adequate solution. Although the fit indices for the six-factor model suggested an acceptable fit to the data, in this model the negative components of the SCS were highly correlated with each other, especially with the over-identification factor.

**Conclusion:**

The results of this study provide evidence to support the use of the separate self-compassion- and self-coldness -scores rather than the overall score of the SCS. Although the fit indices supported the six-factor model, the use of six subscale scores cannot be recommended on the basis of our results given the extremely high correlations within this model between some factors.

## Introduction

Over the past decade, self-compassion has emerged as an important topic of research on human well-being. Self-compassion has been conceptualized as a positive emotional stance towards oneself, in that one extends feelings of kindness and unconditional caring towards oneself [1, 2]. In contrast to *self-esteem*, self-compassion is not based on judgments of one´s characteristics or performance, but puts the focus on feelings of compassion toward oneself and seeing oneself as connected with others by recognition of common humanity [2, 3]. Self-compassion entails three components: 1) kindness and understanding towards oneself rather than self-criticism and judgment; 2) recognition of shared human experience, that is, seeing inadequacies as a part of common humanity rather than feeling isolated by one´s imperfection; and 3) balanced awareness of one´s experience of suffering in moments of distress without overidentifying with negative thoughts and feelings [1–4].

Self-compassion has already shown potential to promote well-being [5] and alleviate suffering [6]. However, a wide majority of the studies on self-compassion have been conducted using the Self-Compassion Scale (SCS) [1], the psychometric validity of which has been recently called into question [7–10]. The SCS was developed using two student samples, and it was designed to measure the three main components of self-compassion on separate subscales (Self-kindness versus Self-judgment, Common humanity versus Isolation, and Mindfulness versus Over-identification). The SCS consists of 26 items (S1 Table), half of which represent the negative ends of three dimensions (i.e., the lack of self-compassion): Self-judgment, Isolation and Over-identification [1].

On the basis of the original studies [1], the SCS was concluded to consist of six factors instead of three (CFI = .91; TLI = .90 in an initial sample; CFI = .93; TLI = .92 in a second sample). In addition to the six-factor model, also a higher order factor model, in which a single higher-order self-compassion factor was explaining the inter-correlations between the six subscale factors, had a marginal fit (CFI = .90; TLI = .88, and, CFI = .92; TLI = .90) [1]. The findings were interpreted as evidence that the six subscales could be either treated separately, or by computing a total score representing the overall level of self-compassion. Although the use of both the individual subscale scores and the total score has been recommended [1, 2, 11], the SCS has been almost exclusively operationalized as the total score, averaged across all 26 items (with the ones representing the negative components reversed).

Although the SCS is the main instrument to assess self-compassion, only few studies have investigated its validity and reliability in adolescent samples. Yet, adolescence represents a time of heightened vulnerability [12] and is, therefore, a period during which examining self-compassion is highly important. Validation of the scale to measure self-compassion in adolescents is a pivotal step in understanding its role among young people.

### Considering alternative conceptualizations of the SCS

Since the publication of the SCS, its factor structure has been subjected to a high level of interest. Studies have yielded inconsistent findings regarding the validity of the six-factor and the higher order factor structures of the scale, reflecting the original conceptualizations of the scale [1].

Although the six-factor structure has been confirmed in several studies [11, 13–20], many studies have failed to replicate this model: In the study of Williams et al. [21] none of the three factor structures tested (the six factor model, a one-factor model, and, a hierarchical model in which the six factors were indicators of an overall self-compassion factor) met the criteria for acceptable model fit. The study of Lopéz et al. [10] suggested that instead of six factors, the SCS consists of two factors, Self-Compassion and Self-Criticism. This two-factor solution, formed by the positively and negatively formulated items respectively, has been supported also by the findings of Costa et al. [7]. Additionally, the recent study of Coroiu et al. [20] provided support for the use of two subscale scores rather than the overall score. The theoretical basis for the two-factor conceptualization is derived from Gilbert’s social mentality theory, according to which the self-soothing aspect of self-compassion taps into the mammalian contentment and safeness system (parasympathetic nervous system), whereas the self-critical response is thought to tap into the threat-defense system (sympathetic nervous system) [22–24, 25].

Findings regarding the higher-order factor structure of the SCS, i.e. the model in which the intercorrelations between the six subscales are explained by one higher order factor, are even more incongruent. The higher-order single factor structure has been replicated in studies of Cunha et al. [16] and Castilho et al. [14] conducted in Portuguese adolescent and adult samples. Support for the higher order factor was found also with a Chinese undergraduate sample [26], and with a Norwegian university student sample [27]. In turn, several studies have failed to provide support for the higher-order single factor structure [19, 21] (in the study of Kotsou and Leys, [18] a weak hierarchical second order structure for the French adaptation of the SCS). In the study of Neff el al. [11], the higher order model showed relatively poor fit across four samples, suggesting it is not representative of the relationship between subscale factors and a general self-compassion factor. However, as a *bifactor* model was shown to have an acceptable fit in the student, community, and meditator samples, it was suggested that an overall self-compassion factor could still be used with some confidence. On a basis of these findings, a bifactor model was introduced as an alternative approach to validly interpret an overall self-compassion score [11]. A bifactor model is a latent structure where each item loads on a general factor and, simultaneously, on one of several group factors [28–29]. The general factor represents what is common among all items. The orthogonal “group” factors, i.e. subscale factors, represent “what is left”, i.e. the part of the variance in item responses which is not accounted for by the general factor [28–29, 30–32].

In the recent in-depth examination of the internal structure of the SCS by Brenner et al. [13], in which eight different models were tested in a sample of 1115 college students, the bifactor model consisting of two general factors—Self-Compassion and Self-Coldness—and six specific factors demonstrated the best fit to the data. Additionally, the results indicated that the Self-Coldness factor accounted for unique variance in depression, anxiety, and stress. In turn, Self-Compassion factor only accounted for unique variance in depression. The results were interpreted as providing support for the presence of two distinct general factors (in addition to the six specific factors); 13 items of the SCS items appeared to contribute to “Self-Compassion”, and the remaining 13 items appeared to contribute to “Self-Coldness” [13].

### The current study

**Fig 1:**
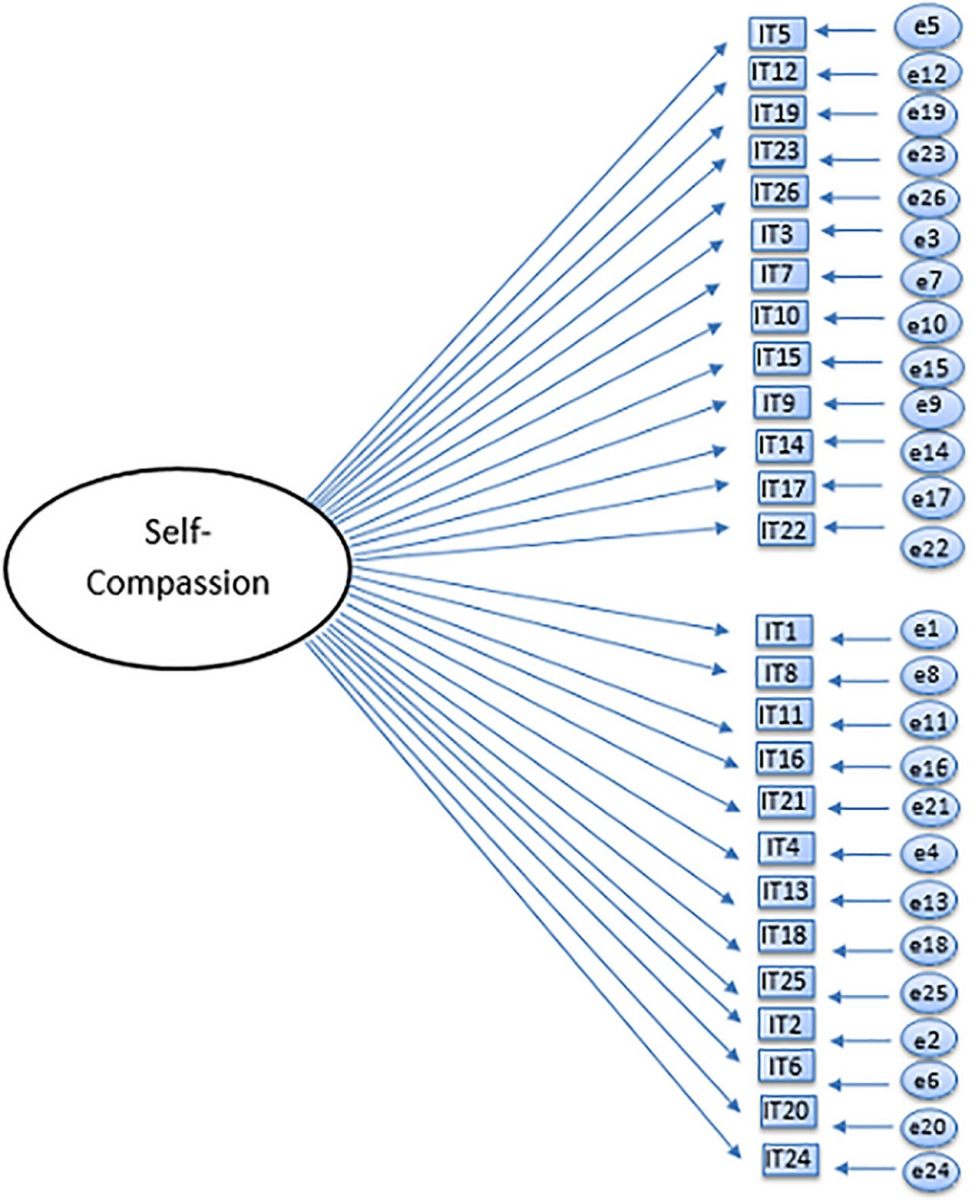
Factor structure of the one-factor model of the Self-Compassion Scale. IT = item, e = error variance.

**Fig 2:**
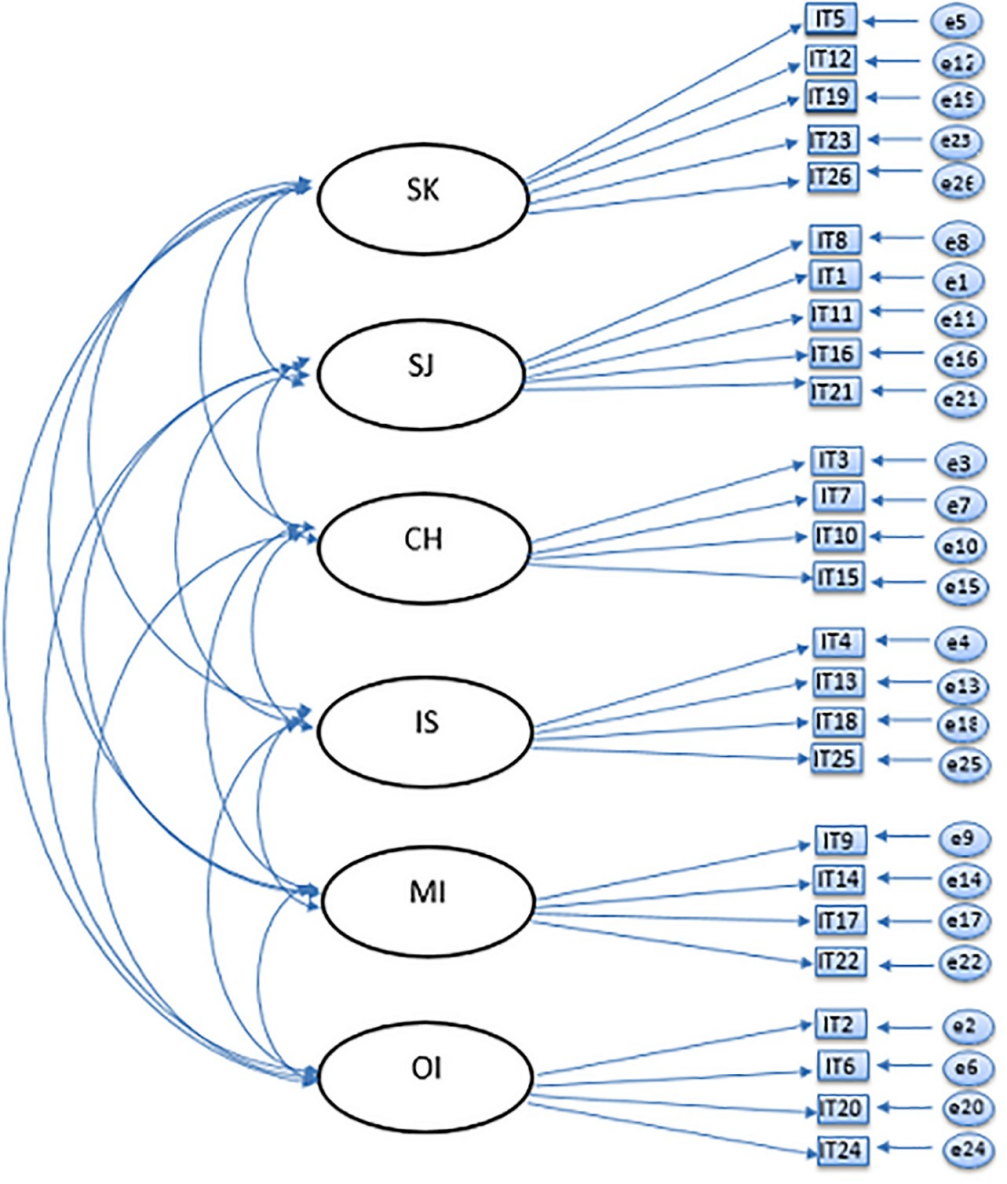
Factor structure of the six-factor model of the Self-Compassion Scale. SK = Self-Kindness; CH = Common Humanity; MI = Mindfulness; SJ = Self-Judgment; IS = Isolation; OI = Over-Identification; IT = item; e = error variance.

**Fig 3:**
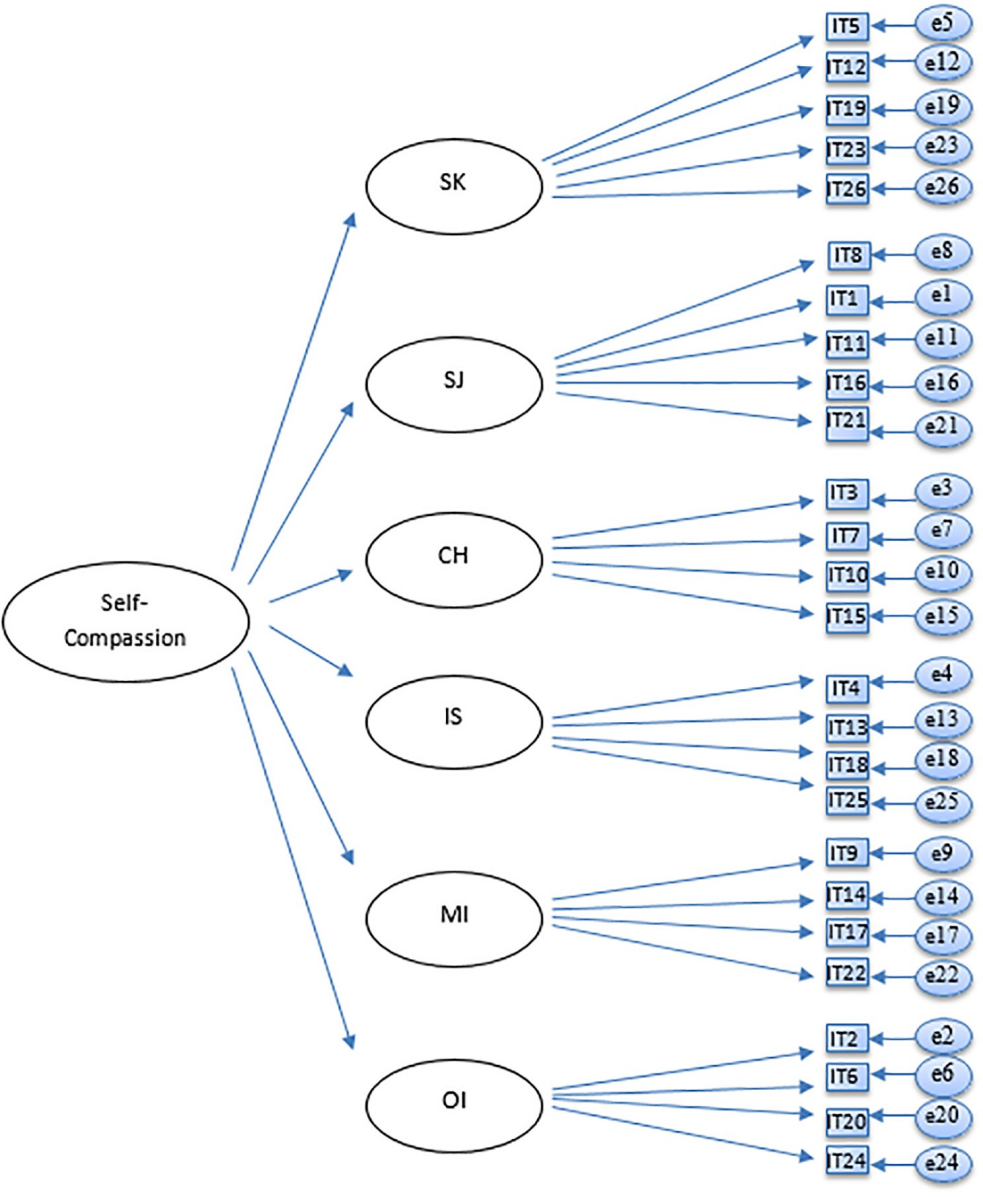
Factor structure of the higher-order model of the Self-Compassion Scale. SK = Self-Kindness; CH = Common Humanity; MI = Mindfulness; SJ = Self-Judgment; IS = Isolation; OI = Over-Identification; IT = item; e = error variance.

**Fig 4:**
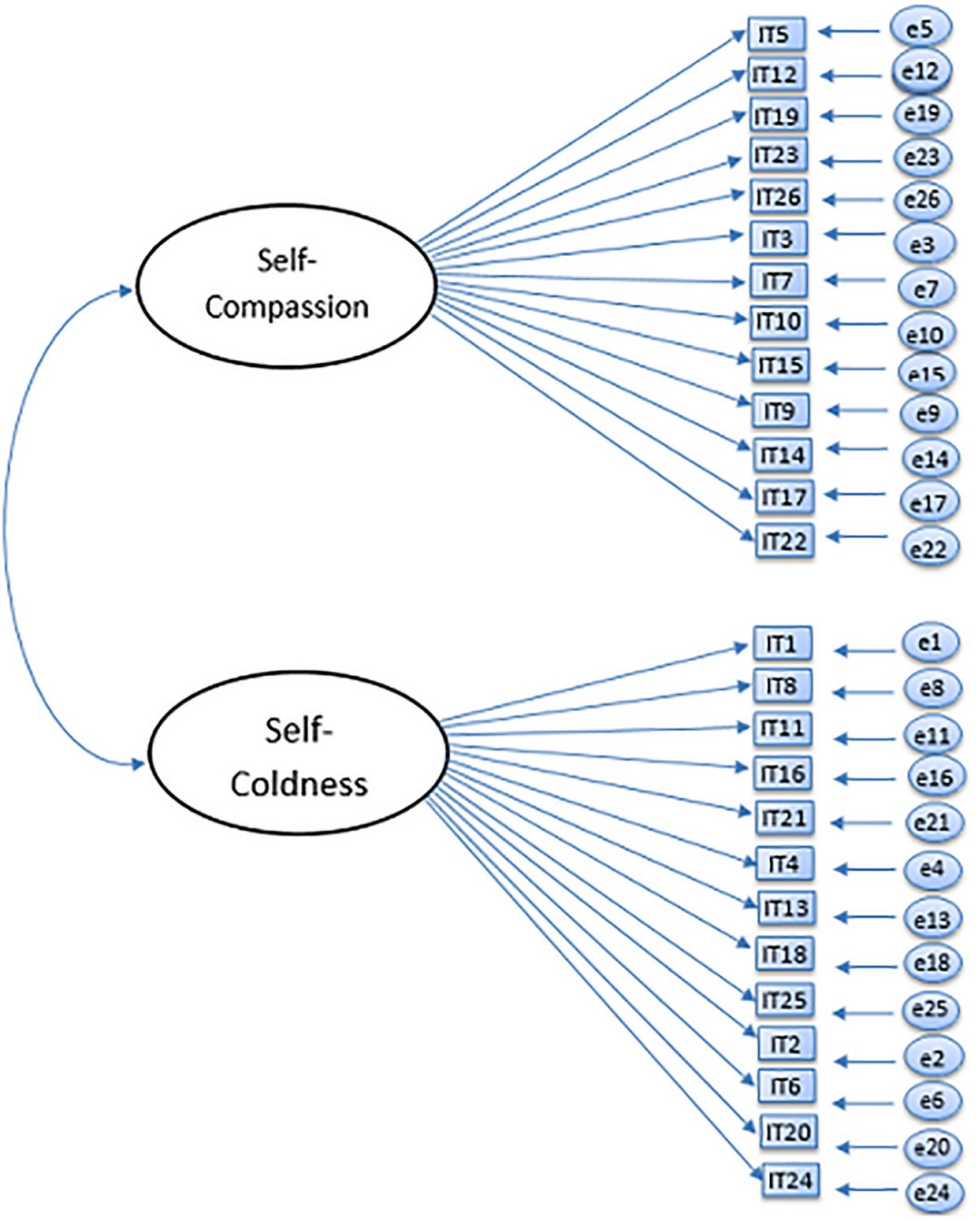
Factor structure of the two-factor model of the Self-Compassion Scale. IT = item; e = error variance.

**Fig 5:**
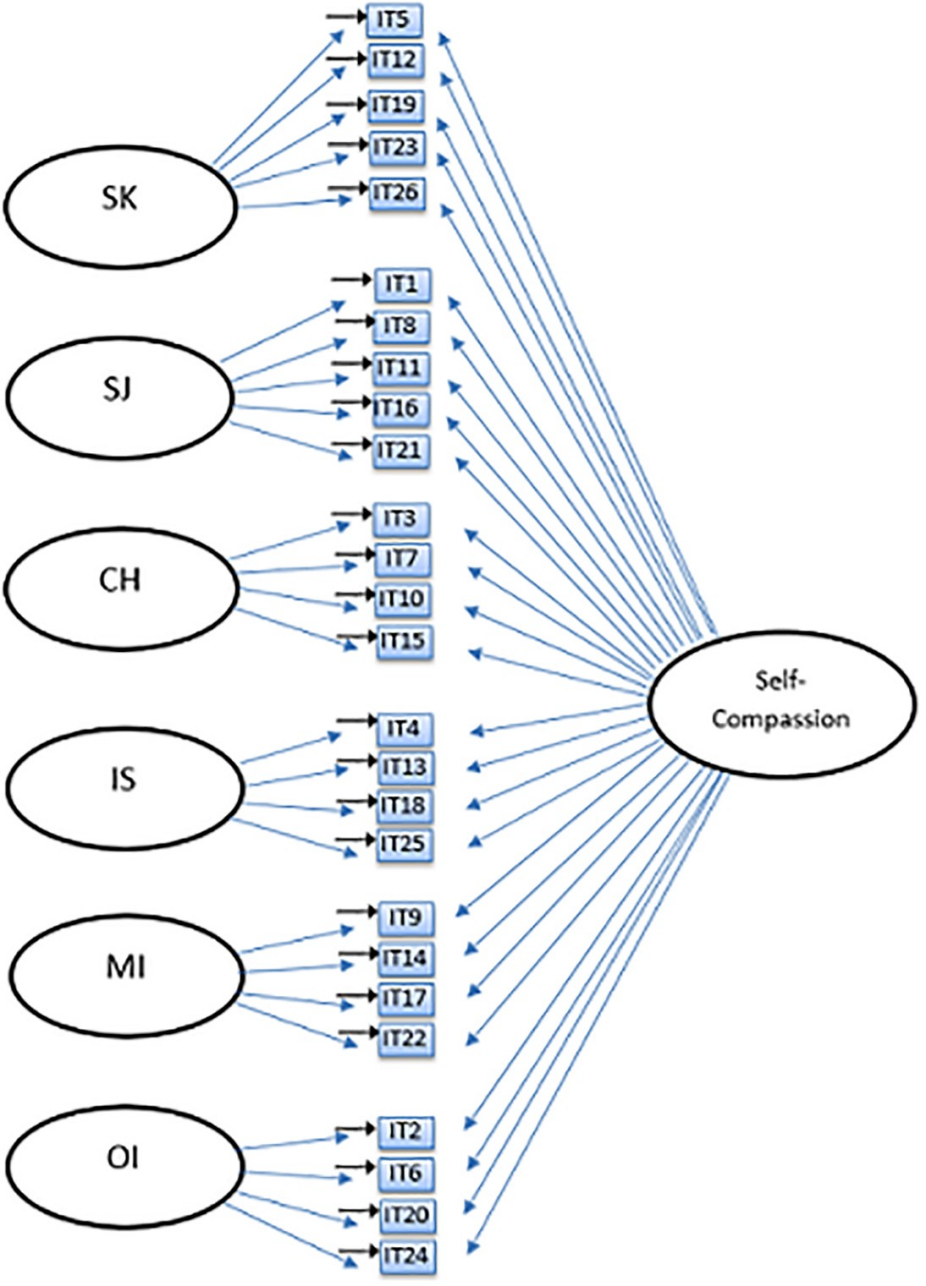
Factor structure of the bi-factor model of the Self-Compassion Scale. SK = Self-Kindness; CH = Common Humanity; MI = Mindfulness; SJ = Self-Judgment; IS = Isolation; OI = Over-Identification; IT = item; e = error variance.

**Fig 6:**
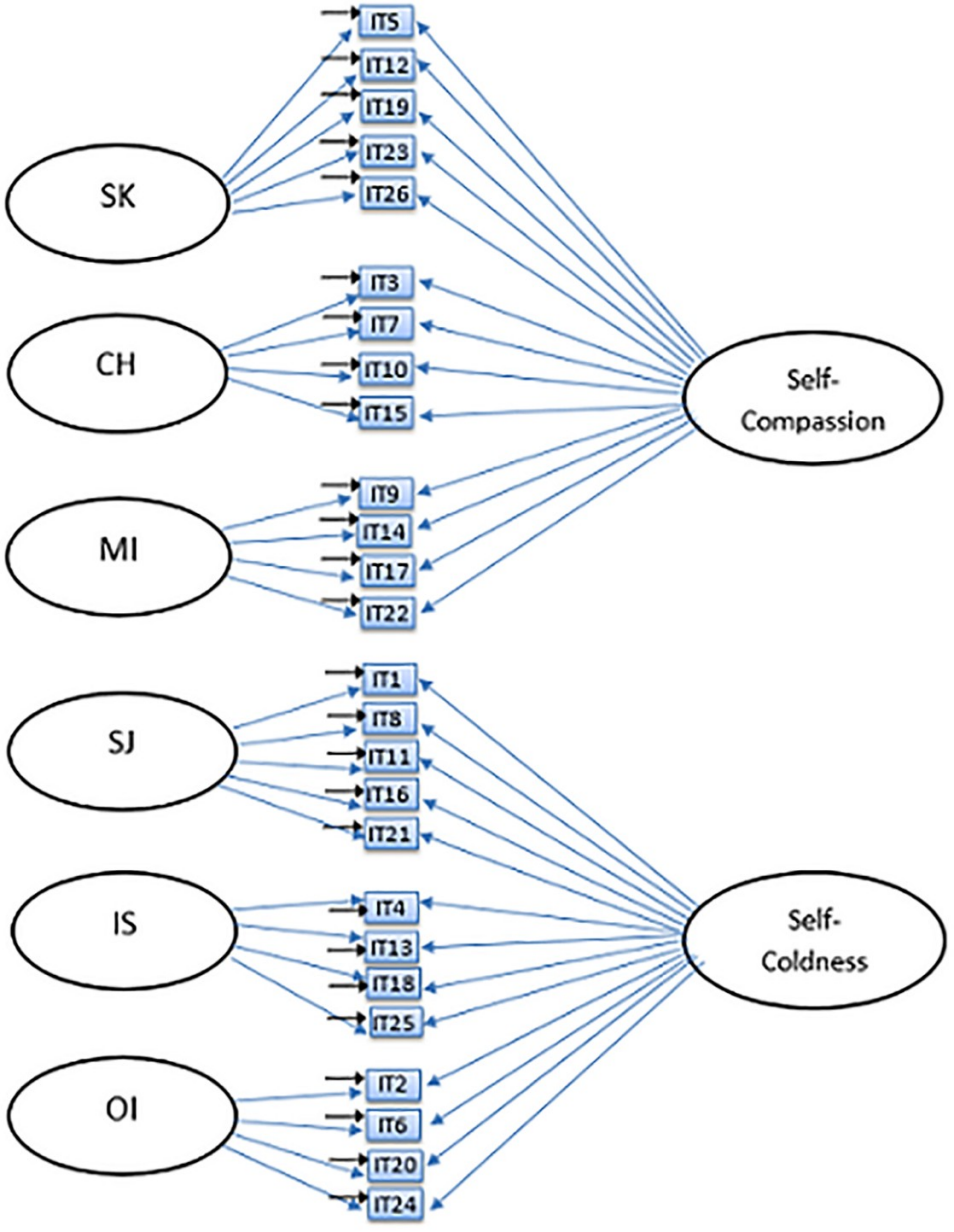
Factor structure of the two-bifactor model of the Self-Compassion Scale. SK = Self-Kindness; CH = Common Humanity; MI = Mindfulness; SJ = Self-Judgment; IS = Isolation; OI = Over-Identification; IT = item.

The current study seeks to examine the factor structure of the Self-Compassion Scale, and in particular, whether the currently used six-factor model and the unidimensional model can be confirmed in a large adolescent sample using confirmatory factor analysis (CFA). Based on previous studies and theory, six different models were tested: a one-factor model (Fig 1), an oblique six-factor model (Fig 2), a higher-order model (Fig 3), an oblique two-factor model (Fig 4), a bifactor model with one general factor and a bifactor model with two general factors (later in this text referred to as bifactor model and two-bifactor model, respectively; Figs 5 and 6).

Additionally, criterion validity [33–34] of the factors of the SCS was evaluated by examining their associations with depression. Based on previous research, self-compassion is inversely related to depression [6–9, 14, 35–37]. In the case of two-dimensionality (i.e., either the two-factor or the two-bifactor model fit the data), the Self-Compassion factor should be negatively, and the Self-Coldness factor positively linked with depression. It was assumed that of the six subscales of the SCS, the positive components would associate inversely and the negative factors positively with depression [13].

## Method

### Participants

The data were collected as a part of a larger project “Well-being for upper secondary education” in two occasions: fall 2016 (wave 1; later referred to as W1, S1 File) and spring 2017 (wave 2; later referred to as W2, S2 File). Data collected in W2 were used for replication purposes. The target sample consisted of 1861 first-year upper secondary school students from eight high schools and eight vocational schools (the latter belonging to three large vocational institutes) in six towns in Southern and South-West Finland. The final sample consisted, at W1, of 1725 students (92.7% of the target sample; 50.3% females, 48.4% males, and 1.3% students who identified themselves as “other”). Large majority (95% of participants) were born either in 1999 or in 2000, i.e. had 15–17 years of age at the time of data collection. W2 data used for replication purposes involved responses from 1497 students (51.3% females, 47.0% males and 1.7% others). They were largely, but not completely, the same students involved in W1 data collection.

### Procedure

Data were collected via web based questionnaires during school hours. Data collection took approximately 45 minutes; the SCS was only a part of a longer questionnaire. Participants were assured confidentiality of their responses and they provided active, informed consent before answering any questions. Lack of guardian consent was accepted by the research ethics committee due to the fact that participants were almost 18 years old, and the current study didn´t involve invasive interventions.

We used planned missingness design [38], which enables researchers to collect incomplete data from participants by randomly assigning participants to have missing items on a survey. This design reduces the burden on participants, and results in smaller rates of unplanned missing data, and consequently higher validity [38–40]. In our study, each student responded to a common set of items, containing e.g., demographics and scales consisting of three or less items, and approximately 67% of the items in other sets (the three item sets were carefully constructed following the guidelines provided in the literature [39, 41–42]. The planned missingness design results in data missing completely at random (MCAR) [38, 43–47]. In order to avoid any bias due to ordering of the questions, the order of the scales in the large questionnaire was randomized into 36 different versions. Each participant answered a version that was randomly assigned to him or her. By using randomized versions of the scales we were able to collect a large amount of data, and the data that was missing was completely at random, resulting in high quality data.

### Measures

#### Self-Compassion Scale (SCS)

Self-compassion was assessed using the Self-Compassion Scale (SCS) [1]. For the purposes of the current study, the SCS questionnaire was translated into Finnish (S1 Table). Two translations were made of the SCS from the original language (English) to the target language (Finnish). The first version of the translation was conducted by an independent professional translator. The second version was made by the research group. After this, the authors discussed the translations in order to discover possible discrepancies reflecting ambiguous wording in the items. With the help of the original questionnaire as well as the translation produced by the independent translator and the translation made by the research group, synthesis of the two translations was finally produced by the authors. To check the validity of this one common translation, and all items were once back-translated by another independent professional, who was totally blind to the original version.

The SCS is a self-report measure that comprises 26 items, 13 of which represent the negative components (i.e., the lack of self-compassion). The items form six subscales of self-compassion: Self-kindness (5 items, e.g. *“I try to be loving towards myself when I´m feeling emotional pain*), Self-judgment (5 items, e.g. *“I´m disapproving and judgmental about my own flaws and inadequacies”*), Common humanity (4 items, e.g. “*When I feel inadequate in some way*, *I try to remind myself that feelings of inadequacy are shared by most people”)*, Isolation (4 items, e.g. “*When I fail at something that´s important to me*, *I tend to feel alone in my failure”*), Mindfulness (4 items, e.g. “*When I´m feeling down I try to approach my feelings with curiosity and openness”)*, and Over-identification (4 items, e.g. “*When I`m feeling down I tend to obsess and fixate on everything that´s wrong“)*.

Responses were given on a 5-point Likert scale ranging from 1 (“hardly ever”) to 5 (“almost always”) (W1), and ranging from 1 (“almost never”) to 5 (“almost always" (W2; the Finnish translations of the Likert scale response options are provided in S1 Table). Items representing the negative components of the SCS are reverse-coded so that higher scores indicate a lower level of the characteristics measured. Subscale scores are computed by calculating the mean of subscale item responses. The total self-compassion score is calculated by computing a grand mean of all six subscale means. In the original paper, internal consistency reliabilities for subscales varied from .75 (Self-kindness) to .81 (Over-identification). Internal consistency for the total score of the 26-item SCS was .92 [1].

#### Revised Beck Depression Inventory (RBDI)

Depression was measured with the Revised Beck Depression Inventory (RBDI; Raitasalo’s modification of the short form of the Beck Depression Inventory, i.e., Mood Questionnaire [48–49]. RBDI consists of 13 items from the original 21–item Beck Depression Inventory [50], which is one of the most widely used self-rating scales for assessing the severity of depression. The RBDI has been validated in Finnish adult population and has shown good internal consistency (α = .92) [48].

In the adolescent population the internal consistency has been reported .84 for girls and .87 for boys [49].

### Analytic approach and assessment of model fit

To analyse the dimensionality of the SCS, we conducted confirmatory factor analyses (CFAs) using statistical software Mplus (v. 7.4; Muthén and Muthén, 2015). Due to the ordinal nature of the item responses and mild violations of multivariate normality, the robust version of maximum likelihood estimation (MLR) was used.

We evaluated the model fit using a variety of indices: χ^2^ -statistic (the Satorra-Bentler scaled chi–square values, referred to as S-B χ^2^ in the Results-section; see also [51]), the standardized root mean square (SRMR), the root mean square of approximation (RMSEA) [52, 53] the Tucker-Lewis Index (TLI; also known as the NNFI) [53], and the Comparative Fit Index (CFI). The issues surrounding guidelines for interpreting goodness-of-fit indices have been vastly debated [54–56]. Based on previous literature, the following model fit criteria were chosen: CFI and TLI ≥ .90 was considered acceptable [53], whereas CFI and TLI ≥ .95 was interpreted as indicative of good model fit [57]. RMSEA values in the range of .06 –.08 were considered acceptable, and values ≤ .06 were considered to indicate good fit [56, 57], SRMR values ≤ .08 were considered acceptable [57], and values ≤ .05 good [58]. In addition, we report the AIC values [59, 60], which can be utilized to compare the relative fit of non-nested models. When modifying the models, we conducted the improvements if they were supported by empirical, conceptual, or practical basis [61, 62]. For reader to evaluate the fit of the models tested in terms of parameter estimates, the fully standardized parameter estimates, standard errors and residual variances of all models (calculated using the W1 data) are provided as supporting information (S2 and S3 Tables). Fully standardized factor loadings, standard errors and residual variances in the preliminary and the final two-factor model, calculated using the W2 data, are also provided as supporting information (S4 and S5 Tables).

### Validity and reliability

The criterion validity of the SCS was evaluated in order to examine whether the components of the SCS are associated with a construct—in our case, depression—as assumed [33, 34]. Based on previous research [6] depression was selected as the criterion variable. Depression was transformed into a latent variable using parcels, which offer advantages over using items [63]. The items were assigned to groups based on their factor loadings, and parcels were then constructed by computing averages of individual items in the group [64]. Reliability was assessed using McDonald’s Omega (ω) since the Cronbach’s alpha (α) tends to underestimate scale reliability if factor loadings vary in magnitude [65], and since it is not recommended to use Cronbach’s alpha when items of the examined scale are multidimensional [66].

## Results

### Preliminary analysis and descriptive statistics

Analyses were first performed (W1) with a sample of 1710 upper secondary school students (15 cases with missing data on all variables were excluded). Descriptive statistics for the SCS total score, subscale scores and RBDI–scores as well as internal reliability coefficients (α and ω) are presented in Table 1. A sample correlation matrix (W1) is shown in Tables 2 and 3. Analyses performed for replication purposes (W2) were conducted with a sample of 1481 students (16 cases with missing data on all variables were excluded). A sample correlation matrix (W2) is shown in Tables 4 and 5.

**Table 1:** Means and standard deviations of the Self-Compassion Scale total score, subscale scores and RBDI scores (W1), and omega coefficient (ω).

Measured variable	M	SD	ω	95% CI
1. SCS total score	3.23	.55	.84	[.81, .86]
2. Self-compassion (13-item)	3.09	.69	.87	[.86, .89]
3. Self-coldness (13-item)	2.63	.81	.91	[.90, .92]
4. Self-kindness	3.08	.83	.80	[.78, .82]
5. Self-judgment	2.63	.93	.85	[.83, .86]
6. Common humanity	2.98	.88	.74	[.71, .77]
7. Isolation	2.54	.94	.80	[.78, .82]
8. Mindfulness	3.20	.84	.73	[.69, .76]
9. Over-identification	2.70	.92	.72	[.69, .75]
10. RBDI	1.77	.57	.88	[.86, .89]

*Note*. M = mean; SD = standard deviation; ω = omega; CI = confidence interval. ωs reported with Bootstrap corrected [BC] 95% CI.

**Table 2:** Standardized correlations of the SCS-items and RBDI-parcels (W1).

Item	5	12	19	23	26	1	8	11	16	21	3	7	10	15	4	13	18	25	9	14	17	22	2	6	20	24	R1	R2
5																												
12	**.51**																											
19	**.43**	**.48**																										
23	**.36**	**.38**	**.46**																									
26	**.44**	**.37**	**.46**	**.61**																								
1	**-.16**	**-.13**	**-.26**	**-.41**	**-.30**																							
8	**-.10**	-.06	**-.33**	**-.23**	**-.18**	**.48**																						
11	-.05	.04	**-.10**	**-.23**	**-.23**	**.45**	**.45**																					
16	**-.10**	-.04	**-.26**	**-.29**	**-.20**	**.48**	**.63**	**.49**																				
21	**-.12**	-.04	**-.31**	**-.27**	**-.20**	**.47**	**.61**	**.46**	**.60**																			
3	**.32**	**.29**	**.25**	**.27**	**.33**	.02	.07	.03	**.14**	-.02																		
7	**.34**	**.31**	**.24**	**.18**	**.24**	.05	**.14**	**.13**	**.12**	**.15**	**.38**																	
10	**.36**	**.32**	**.29**	**.20**	**.23**	-.00	.05	**.07**	.07	.05	**.35**	**.64**																
15	**.42**	**.42**	**.41**	**.46**	**.49**	**-.19**	-.06	-.06	-.04	-.07	**.46**	**.34**	**.34**															
**4**	-.05	**-.07**	**-.17**	**-.23**	**-.18**	**.42**	**.44**	**.36**	**.37**	**.43**	.01	**.16**	.07	-.08														
13	**-.10**	.01	**-.14**	**-.18**	**-.13**	**.41**	**.44**	**.37**	**.42**	**.49**	-.02	**.11**	.09	-.07	**.47**													
18	**-.11**	-.05	**-.11**	**-.19**	-.11	**.34**	**.38**	**.32**	**.32**	**.43**	-.05	.08	.06	-.07	**.47**	**.62**												
25	**-.15**	**-.11**	**-.21**	**-.22**	**-.14**	**.43**	**.42**	**.39**	**.43**	**.51**	-.04	.02	-.03	**-.13**	**.46**	**.49**	**.45**											
9	**.30**	**.20**	**.18**	**.25**	**.30**	.01	.10	.06	**.10**	.07	**.32**	**.16**	**.20**	**.29**	.02	.03	-.02	-.00										
14	**.33**	**.41**	**.26**	**.30**	**.36**	-.00	.13	.05	**.12**	.06	**.38**	**.27**	**.32**	**.45**	.01	.00	-.02	-.06	**.35**									
17	**.36**	**.37**	**.30**	**.35**	**.39**	**-.12**	.01	.02	.03	.05	**.31**	**.25**	**.32**	**.46**	-.05	-.04	-.04	**-.15**	**.39**	**.48**								

*Note*. SCS-items referred to as [number], and RBDI-parcels referred to as R[number]. Correlations which were significant at the *p≤* .001 level are bolded.

**Table 3:** Standardized correlations of the SCS-items and RBDI-parcels (W1).

Item	5	12	19	23	26	1	8	11	16	21	3	7	10	15	4	13	18	25	9	14	17	22	2	6	20	24	R1	R2
22	**.39**	**.40**	**.36**	**.37**	**.40**	-.06	.01	**.01**	.00	.00	**.35**	**.31**	**.28**	**.41**	-.05	-.03	-.03	-.11	.24	**.44**	**.39**							
2	**-.14**	**-.09**	**-.22**	**-.34**	**-.19**	**.59**	**.52**	**.42**	.48	**.51**	.03	**.12**	.02	-.11	**.51**	**.44**	**.43**	**.45**	-.01	.03	-.11	**-.09**						
6	**-.07**	**-.03**	**-.19**	**-.24**	**-.11**	**.46**	**.52**	**.36**	.45	**.47**	.05	**.18**	.10	-.08	**.60**	**.43**	**.40**	**.48**	.04	.03	-.06	**-.06**	**.48**					
20	**.06**	**.12**	-01	-.09	-.03	**.29**	**.31**	**.39**	.31	**.51**	.08	**.23**	**.14**	.07	**.37**	**.39**	**.41**	**.44**	-.12	.09	-.05	**.11**	**.41**	**.4**				
24	.04	.07	-.01	-.10	-.10	**.23**	**.31**	**.32**	.35	**.34**	-.02	.07	.10	.01	**.31**	**.36**	**.39**	**.40**	-.02	.00	-.04	.02	**.34**	**.31**	**.43**			
R1	**-.27**	**-.18**	**-.30**	**-.35**	**-.26**	**.50**	**.36**	**.33**	.37	**.40**	**-.10**	**-.10**	**-.11**	**-.21**	**.39**	**.46**	**.35**	**.44**	**-.08**	-.11	**-.16**	**-.21**	**.45**	**.31**	**.27**	**.24**		
R2	**-.24**	**-,24**	**-.29**	**-.30**	**-.23**	**.43**	**.32**	**.25**	.30	**.40**	**-.10**	**-.08**	**-.12**	**-.22**	**.38**	**.40**	**.32**	**.38**	**-.08**	-.07	**-.16**	**-.16**	**.40**	**.33**	**.25**	**.17**	**.62**	
R3	**-.25**	**-.17**	**-27**	**-.30**	**-.22**	**.43**	**.32**	**.30**	.31	**.39**	**-.13**	**-.08**	**-.09**	**-.19**	**.39**	**.43**	**.32**	**.38**	**-.07**	**-.11**	**-.16**	**-.18**	**.43**	**.30**	**.25**	**.28**	**.77**	**.66**

*Note*. SCS-items referred to as [number], and RBDI-parcels referred to as R[number]. Correlations which were significant at the *p≤* .001 level are bolded.

**Table 4:** Standardized correlations of the SCS-items and RBDI-parcels (W2).

Item	5	12	19	23	26	1	8	11	16	21	3	7	10	15	4	13	18	25	9	14	17	22	2	6	20	24	R1	R2
5																												
12	**.55**																											
19	**.48**	**.46**																										
23	**.46**	**.47**	**.50**																									
26	**.47**	**.48**	**.54**	**.65**																								
1	**-.12**	-.09	**-.18**	**-.26**	**-.16**																							
8	-.04	.03	**-.22**	**-.19**	**-.18**	**.55**																						
11	-.03	.04	**-.13**	**-.18**	**-.15**	**.52**	**.45**																					
16	-.05	.03	**-.17**	**-.22**	-.09	**.57**	**.65**	**.57**																				
21	-.11	.03	**-.31**	**-.12**	-.05	**.47**	**.60**	**.47**	**.61**																			
3	**.46**	**.36**	**.26**	**.39**	**.36**	.08	**.17**	.07	**.17**	.07																		
7	**.49**	**.38**	**.24**	**.26**	**.32**	.04	**.12**	.05	.09	.09	**.43**																	
10	**.45**	**.35**	**.30**	**.27**	**.34**	.03	**.10**	**.23**	**.13**	.07	**.39**	**.66**																
15	**.51**	**.52**	**.44**	**.51**	**.53**	-.07	-.09	-.01	.05	.01	**.50**	**.43**	**.46**															
4	.03	.04	**-.10**	**-.14**	-.03	**.53**	**.45**	**.40**	**.45**	**.49**	.11	**.19**	**.16**	.00														
13	-.06	.18	**-.09**	-.08	-.03	**.44**	**.49**	**.47**	**.51**	**.50**	.08	**.13**	**.16**	.05	**.51**													
18	-.00	.03	-.04	-.06	.00	**.38**	**.45**	**.32**	**.43**	**.44**	.05	.11	**.14**	.00	**.49**	**.67**												
25	-.04	-.01	**-.13**	-.09	-.05	**.47**	**.46**	**.39**	**.47**	**.54**	.07	.06	**.10**	.00	**.55**	**.45**	**.49**											
9	**.36**	**.31**	**.32**	**.33**	**.41**	.10	**.17**	**.13**	**.16**	**.13**	**.45**	**.34**	**.31**	.37	.06	.07	-.01	.06										
14	**.38**	**.45**	**.26**	**.37**	**.42**	.10	**.20**	.19	**.21**	**.19**	**.42**	**.42**	**.39**	.57	.11	**.18**	.10	.07	**.47**									
17	**.40**	**.48**	**.43**	**.44**	**.49**	.09	.06	.06	**.14**	.10	**.40**	**.37**	**.39**	.58	.02	.07	.05	.05	**.46**	**.51**								

*Note*. SCS-items referred to as [number], and RBDI-parcels referred to as R[number]. Correlations which were significant at the *p≤* .001 are bolded

**Table 5:** Standardized correlations of the SCS-items and RBDI-parcels (W2).

Item	5	12	19	23	26	1	8	11	16	21	3	7	10	15	4	13	18	25	9	14	17	22	2	6	20	24	R1	R2
22	**.42**	.**50**	**.47**	**.47**	**.50**	-.06	-.01	.02	.04	.03	**.38**	**.32**	**.39**	.49	-.03	-.01	-.02	-.03	**.34**	**.50**	.**51**							
2	-.05	-.03	**-.18**	**-.21**	**-.12**	**.64**	**.54**	**.51**	**.54**	**.50**	.08	.08	.09	-.06	**.60**	**.52**	**.45**	**.51**	**.08**	.08	-.05	**-.13**						
6	.06	.05	**-.12**	**-.17**	-.05	**.59**	**.59**	**.45**	**.54**	**.49**	**.17**	**.13**	.13	-.03	**.63**	**.51**	**.44**	**.55**	**.12**	**.16**	.00	-.05	**.63**					
20	.**15**	**.24**	-.08	.05	.05	**.34**	**.37**	**.38**	**.41**	**.47**	**.13**	**.21**	.21	**.12**	**.39**	**.44**	**.42**	**.41**	-.00	**.18**	.06	**.16**	**.44**	**.42**				
24	.09	**.13**	-.04	-.01	.03	**.33**	**.37**	**.34**	**.33**	**.37**	.08	**.11**	.14	.03	**.36**	**.42**	**.43**	**.48**	-.01	**.14**	.03	.06	**.46**	**.38**	**.43**			
1	**-.20**	**-.16**	**-.21**	**-.24**	**-.19**	**.50**	**.39**	**.34**	**.36**	**.42**	-.08	-.09	-.06	**-.17**	**.40**	**.44**	**.35**	**.45**	-.03	-.03	**-.11**	**-.17**	**.48**	**.44**	**.31**	**.26**		
2	**-.18**	**-.21**	**-.25**	**-.24**	**-.20**	**.42**	**.33**	**.29**	**.33**	**.42**	-.06	-.10	-.11	**-.18**	**.41**	**.39**	**.28**	**.41**	-.03	-.04	**-.13**	**-.20**	**.45**	**.37**	**.25**	**.22**	**.66**	
3	**-.20**	**-.17**	**-22**	**-.25**	**-.19**	**.44**	**.36**	**.32**	**.32**	**.42**	-.07	-.11	-.09	**-.17**	**.39**	**.42**	**.32**	**.38**	-.14	-.04	**-.13**	**-.18**	**.43**	**.40**	**.26**	**.22**	**.77**	**.67**

*Note*. SCS-items referred to as [number], and RBDI-parcels referred to as R[number]. Correlations which were significant at the *p≤* .001 are bolded.

### Confirmatory factor analysis

The fit indices for the six models tested (preliminary and final) are presented in Table 6. All models were re-tested with the data collected at W2: the results found with W1 data were largely replicated (Table 7).

**Table 6:** Fit indices for confirmatory factor analyses of the models tested (W1).

Model	S-B χ^2^	df	CFI	TLI	RMSEA[90%Cl]	SRMR	AIC	Comparison With Model 3		Comparison With Model 6	
								Δ χ^2^	Δdf	Δ χ^2^	Δ df
1.One-factor model	5089.00	299	.52	.48	.09 [.08, .09]	.17	84658.07	Not nested		Not nested	
2.Preliminary six-factor model	1299.05	284	.91	.89	.04 [.04, .04]	.07	80898.14	104.70*	2	Not nested	
3.Final six-factor model	1114.20	282	.92	.91	.04 [.03, .04]	.06	80717.27			Not nested	
4.Higher-order model	2785.77	293	.76	.73	.06 [.06, .06]	.14	82366.76	Not nested		Not nested	
5.Preliminary two-factor model	1942.83	298	.84	.83	.05 [.05, .05]	.09	81513.87	Not nested		333.91*	7
6.Final two-factor model	1387.20	291	.90	.88	.04 [.04, .04]	.08	80972.22	Not nested			
7.Bifactor model**	-	-	-	-	-	-	-	-		-	
8.Two-bifactor model	1326.08	273	.90	.88	.04 [.04, .05]	.09	80947.07	Not nested		Not nested	

*Note*. N = 1710. S-B χ^2^ = Satorra-Bentler scaled chi-square values; df = degrees of freedom; CFI = comparative fit index; NNFI = non-normed fit index; RMSEA = root mean square error of approximation; SRMR = standardized root mean square residual; AIC = Akaike information criterion. Final six-factor model had 2 correlating residuals. Final two-factor model had 7 correlating residuals. Bifactor model had one fixed residual. *Not nested* indicates that the model is not compared to Model 3, or Model 6.

*p < .001.

** Did not converge.

**Table 7:** Fit indices for confirmatory factor analyses of the models tested (W2).

Model	S-B χ^2^	df	CFI	TLI	RMSEA [90%Cl]	SRMR	AIC	Compariso with Model 3		Comparison with Model 6	
								Δ χ^2^	Δ df	Δ χ^2^	Δ df
1.One-factor model	7091.50	299	.35	.29	.11[.11, .11]	.23	73649.01	Not nested		Not nested	
2.Preliminary six-factor	1312.40	284	.91	.90	04 [.04, .04]	.07	67899.97	78.85*	2	Not nested	
3.Final six-factor model	1156.76	282	.93	.92	.04 [.03, .04]	.06	67750.98			Not nested	
4. Higher order model	3157.88	293	.74	.71	.07 [.07, .07]	.17	69727.43	Not nested		Not nested	
5. Preliminary two-factor model	1840.00	298	.87	.85	05 [.05, .05]	.08	68399.54	Not nested		340.68*	7
6. Final two-factor model	1377.52	291	.91	.90	.04 [.04, .04]	.08	67951.10	Not nested			
7. Bifactor model	2888.35	274	.76	.72	.07 [.07, .07]	.17	69496.00	Not nested		Not nested	
8.Two-bifactor model**	1288.08	274	.91	.89	.04 [.04, .05]	.08	67895.62	Not nested		Not nested	

*Note*. N = 1482, S-B χ^2^ = Satorra-Bentler scaled chi-square values; df = degrees of freedom; CFI = comparative fit index; NNFI = non-normed fit index; RMSEA = root mean square error of approximation; SRMR = standardized root mean square residual; AIC = Akaike information criterion. Final six-factor model had 2 correlating residuals. Final two-factor model had 7 correlating residuals.

*Not nested* indicates that the model is not compared to Model 3, or Model 6.

*p < .001.

**The model did not converge until we fixed the variance of item 9 to zero.

#### One-factor model

The first model with all items loading on a single factor did not fit our data (Tables 6 and 7). Factor loading estimates (W1) varied from .01 (item 10) to .74 (item 8) (i.e. range of *R*^2^s = .00–55; range of *p*s = .00 –.83).

#### Six-factor model

The preliminary CFA results indicated an adequate fit of the six-factor model (Tables 6 and 7). All items (in both waves) loaded significantly on to their purported factors (*p* = .00). The loadings (W1) varied from .50 (item 24 on Over-identification) to .78 (item 8 on Self-judgment). Residuals varied from .42 (item 16) to .76 (item 24). Over-identification factor had very high correlations with other two negative factors: *r* = .94 and *r* = .98 (both *p*s = .00) with Self-judgment, and Isolation, respectively. Modification indices suggested that adding residual correlations between several pairs of items would be of benefit. Two residual correlations between items that loaded on the same factor were allowed (items 7 and 10, loading on Common humanity; items 13 and 18, loading on Isolation).

The respecified six-factor model with two residual correlations fit slightly better to our data (Table 6). Factor loading estimates revealed that the indicators were significantly related to their purported factors (range of *R*^2^s = .24 (item 24)–.61 (item 8), *p*s = .00). As in the preliminary model, the Over-identification factor was highly correlated with two other negative factors (*r* = .93, and *r* = .98 with Self-judgement and Isolation, respectively; *p*s = .00). Correlation between Self-judgement and Isolation was .83 (*p* = .00). Correlations among the positive factors were as follows: *r* = .81 (correlation between Self-kindness and Common humanity), *r* = .86 (correlation between Common humanity and Mindfulness) and *r* = .78 (correlation between Self-kindness and Mindfulness), (*p*s = .00). This modified model provided a significantly superior fit to the data than the original six-factor solution in both waves (Tables 6 and 7).

#### Higher-order factor model

Adding a higher order factor to the six-factor model deteriorated the fit of the model. Fit indices for the higher-order model showed an unsatisfactory fit (Tables 6 and 7). Factor loading estimates for the six subscales (W1) varied from .49 (item 24) to .86 (item 7), all *p*s = .00. However, the six subscales did not load onto the higher order factor as expected: although Self-kindness and Mindfulness loaded positively (and weakly) onto the higher order factor (.32, *p* = .00, and .01, *p* = .78, respectively), Common humanity had a negative loading onto the higher order factor (–.12, *p* = .01). In turn, factors of negative valence (reverse items used) loaded very strongly onto the higher order factor: factor loading estimates were .91 for Self-judgment, and .90 for Isolation (both *p*s .00). The Over-identification factor had a standardized loading > 1.00 (1.04) and a negative residual variance < .00 (–.06), indicating that the model was somehow poorly specified and not supported by the data. In addition, factor Over-identification had very high correlations with two other negative factors. Overall, higher-order factor model did not fit the data.

#### Two-factor model

Before any respecifications, the goodness-of-fit indices for the two-factor model indicated an unsatisfactory fit (Tables 6 and 7). The respecified model, with seven residual correlations between items 7 and 10, items 26 and 23, items 9 and 17 (loading on to the Self-Compassion factor) and items 16 and 8, items 18 and 13 items 1 and 2, and finally, items 4 and 6 (loading on to the Self-Coldness factor) showed a better fit (Tables 6 and 7). These improvements, suggested by the modification indices, were justified on the basis that items, the residuals of which were allowed to correlate, loaded on the same factor (items 7, 10, 26, 23, 9 and 17 on Self-Compassion; items 16, 8, 18, 13, 1, 2, 4 and 6 on Self-Coldness; see, S2, S3, S4 and S5 Tables).

Factor loadings of the Self-Compassion items (W1) ranged from .42 (item 9) to .71 (item 15). Factor loadings of the Self-Coldness items ranged from .50 (item 24) to .74 (item 21). Factor loadings of all items were statistically significant (*p*s = .00; range of *R*^2^s = .20–54). The correlation between the two factors, Self-Compassion and Self-Coldness, was negative (*r* = –.14; *p* = .00). The standardized residuals ranged from .45 (item 21, *p* = .00) to .82 (item 9, *p* = .00). No other localized areas of strain were discovered, and this modified two-factor model had a significantly better fit to the data than the original unmodified preliminary model in both waves (Tables 6 and 7). Overall, the fit indices for the final two-factor model indicated an adequate fit.

#### Bifactor approach

The preliminary version of the third model tested, the bifactor model, did not converge at all (W1), or had a very poor fit (W2; Table 7). Factor loading estimates (for the subscales in W2) varied from–.04 (p = .71; item 6 on Over-identification) to .81 (item 7 on Common humanity). Item loading estimates onto general factor varied from -.21 (p = .00; item 14) to .81 (p = .00; item 2).

Finally, we examined a two-bifactor model where all 26 items loaded on one of two general factors (Self-Compassion and Self-Coldness), and, simultaneously on one of six group factors. To set the metric, all factor loadings were freed and the variances of all factors were fixed to one [61]. The two-bifactor model showed a marginal fit in both waves (Tables 6 and 7. However, in this model (W1) item 24 had a strong negative residual variance (-12.06) and a fully standardized factor loading estimate <1 (3.58). Additionally, the two-bifactor model (W2) did not converge until we fixed the residual variance of item 9 to zero.

#### Reliability analysis

As shown in Table 1, the omega coefficients for the six subscales varied from .72 (Over-identification; adequate) to .85 (Self-judgment; very good). Omegas were .84 for the total score, .87 for Self-Compassion (13 items), and .91 for Self-Coldness (13 items), all indicating good reliability.

#### Criterion validity

To test criterion validity, Pearson’s correlation coefficients were calculated between both the six components of the SCS and depression as well as between the two components (13-item Self-Compassion and 13-item Self-Coldness) and depression. These two models were selected on the basis of results of confirmatory factor analyses, which had indicated satisfactory or good fit of these models.

Depression was transformed into a latent variable using parcels. Parcel 1 was constructed by computing averages of BDI items 6 (standardized factor loading .80), 10 (.68), 2 (.67), 9 (.48), and 8 (.48); parcel 2 of BDI items 1 (.78), 3 (.72), 7 (.59), and 11 (.49); and, parcel 3 of items 5 (.78), 4 (.77), 12 (.53), and 13 (.53). Self-judgment, Isolation, and Over-identification were positively correlated with depression (*r* = .56, *r* = .64, and *r* = .53, respectively). In turn, Self-kindness, Common humanity and Mindfulness correlated negatively with depression (*r* = –.47, *r* = –.29, and *r* = –.29, respectively). In the context of the two-factor model, Self-Coldness was positively (*r* = .60), and Self-Compassion negatively (*r* = –.40) associated with depression.

## Discussion

As recent research has provided inconsistent findings regarding whether self-compassion can be reliably and validly assessed by its six components, or by using an overall score [7, 10–11, 13–21], the current study examined the psychometric features of the SCS in an adolescent sample. The internal structure of the SCS was evaluated by testing six models: a one-factor model, an oblique six- factor model, a higher-order model, an oblique two-factor model, a bi-factor model with one general factor (bifactor model) and a bi-factor model with two general factors (two-bifactor model). Criterion validity of the measure was evaluated by examining the associations between different factors of the SCS and depression.

Though the descriptive goodness-of-fit indices and the smallest AIC-value supported the six-factor model, more thorough inspection revealed that there were high correlations among the negative components. Especially the Over-identification factor had a considerably strong association with both Self-judgment and Isolation (.93, and .98, respectively). Also the positive components of the SCS (Self-kindness, Common humanity and Mindfulness) were strongly associated with each other. This leads into the question to what extent these factors measure separate constructs.

The high correlations among the positive factors, and among the negative factors, found in the present study, indicates strong multicollinearity, and raises the question: Are the six subscales really separate from each other, or, should the three negative subscales, as well as the three positive subscales, be combined, resulting in two separate subscales, Self-Compassion and Self-Coldness (see also, studies e.g., [7, 10, 13])? Contrary to the original presentation of the SCS [1, 11, 13, 14, 16–18, 20], our results imply that the six-factor structure may not be advocated for use on the on the ground of poor discriminant validity.

Moreover, our results call into question the validity of the unidimensional approach to the SCS. The models reflecting the unidimensional approach to self-compassion (one-factor model, higher order model, bifactor model) did not fit to our data. However, the two-factor model had an acceptable fit with data from both waves. Only a small correlation (*r* = –.14, *p* = .00) between the two factors, Self-Compassion and Self-Coldness, was discovered indicating that they represent two distinct constructs rather than opposite ends of a continuum. Additionally, the two-bifactor model provided a marginal fit in both samples, suggesting the existence of two general factors rather than one. However, the findings regarding the fit of the two-bifactor model should be interpreted with caution given that the lack of convergence in W2 indicated that the model might not fit the data well. Overall, these results provide support for the presence of two separate constructs instead of one overall construct of self-compassion.

The results of the present study supports the arguments of Gilbert and colleagues, according to whom the positive and negative self-affect should not be represented by a unidimensional scale score [67]. In our study, it was found that the negative factors loaded much more strongly onto the higher order factor than the positive factors. As the negative indicators have been shown to have significantly stronger links to mental health problems than the positive indicators [36], our findings provide support for the idea that the use of a total self-compassion score of the SCS might result in an inflated relationship with symptoms of psychopathology, as suggested by Muris and Petrocchi [36].

Additionally, one can argue that the current results indicating two-dimensionality of the SCS is in congruence with the idea of self-compassion tapping into the mammalian caregiving system, which is associated with the parasympathetic nervous system, and, self-criticism (in our study referred as Self-Coldness) tapping into the threat-defense system, which is associated with the sympathetic nervous system [25, 67, 68]. (However, Neff et al. refer to Engen and Singer, 2016, noting that Self-kindness and Mindfulness are likely to tap into differing neurological and physiological systems; see [11] and references therein). However, arguments regarding the neurophysiology of self-compassion are highly speculative, since, to date, very little research has been conducted on the topic.

In terms of the criterion validity of the scale, results showed that the positive subscales of self-compassion were negatively associated with depression, and the negative subscales had a positive link to this attribute, confirming the validity of the scales. Self-Coldness was more strongly associated with depression (*r* = .60, *p* = .00) than Self-Compassion (*r* = –.40, *p* = .00). These findings are in accordance with the previous studies which have indicated that the negative components of the SCS are more related to psychopathology than the positive components of Self-kindness, Common humanity, and Mindfulness [13, 36]. It should be pointed out that the correlations do not say anything about a possible direction of effects, or whether a third variable accounts for the correlation. The causal relationship between the factors of the SCS and depression should be studied in the future in a longitudinal design.

Overall, the results of the current study suggest that when utilizing the SCS, one should prefer the two-dimensional approach, using the scores of positive and negative components separately. The use the six subscale scores as independent indicators of the components of self-compassion, or the use of a total score, can not be recommended on the basis of our results. However, it should be noted that the two-factor model (and two-bifactor model) had only a marginal fit to the data in the current study. Due to the inconsistent findings concerning the two-dimensional nature of the SCS, and given that the SCS is currently the most widely used measure to assess self-compassion, more research is needed.

### Limitations and future directions

Given that the current study was conducted in a sample consisting of adolescents, the findings may not be applicable to other age groups. Since the results are inconsistent with findings of Cunha et al. [16], a study also conducted in an adolescent sample, more research is needed in order to validate the factor structure of the SCS among people transitioning from childhood to adulthood, and across gender and time. Studies of specifically defined groups, such as adolescents, are important and may extend our knowledge [69] and, in the context of self-compassion, may provide valuable information on whether and how the structure of self-compassion varies across age groups. Since the majority of the studies investigating the psychometric properties of the SCS have been conducted in adult populations, there is an eminent need of examining the internal structure of the scale especially in adolescents, given that the SCS is recommended as an appropriate measure for ages 14 and up.

Additionally, it is important to acknowledge that although our results don´t provide support for assessing self-compassion by its six components, it is possible that the very high correlations between the factors of a same valence (i.e., strong associations among positive factors, and, strong associations among negative factors) may derive from the nature of the sample of this study: it is possible, that the participants, consisting of adolescents from upper secondary and vocational school, have been able to distinguish between the positive and negative items, but not between more nuanced subscale differences. Given the age of participants (approximately 16.5 years), it might be that many of the questions have been hard to grasp since the questions of the SCS require participants to reflect on their own mind and experience in a way and scope that may not be very familiar or easy for at least some of them. Further research on the factor structure of the SCS conducted in other adolescent samples would be of benefit.

Future studies could also investigate the SCS using exploratory SEM bifactor analysis, as recently suggested [70], which may help to contribute to a better understanding of the construct of self-compassion. It is crucial to know what it is that we actually measure when we use the SCS. Validating the measure is an essential step towards studying self-compassion and gaining insight into its potential to enhance mental health and to alleviate suffering.

## Supporting information

S1 Table Items of the Self-Compassion Scale. Items translated into Finnish (F) used in the current study are provided in italics. Subscales of each item are provided in parentheses.
(PDF)

S2 Table Fully standardized factor loadings, standard errors and residual variances in the preliminary two-factor model (W1). (PDF)

S3 Table Fully standardized factor loadings, standard errors and residual variances in the two-factor model with seven residual correlations (W1). (PDF)

S4 Table Fully standardized factor loadings, standard errors and residual variances in the preliminary two-factor model (W2). (PDF)

S5 Table Fully standardized factor loadings, standard errors and residual variances in the two-factor model with seven residual correlations (W2). (PDF)

S1 File W1 data. (SAV)

S2 File W2 data. (SAV)
