# Supplementary material for: Two is more valid than one, but is six even better? The factor structure of the Self-Compassion Scale (SCS)
Source: PLoS One. 2018 Dec 5;13(12):e0207706. doi: 10.1371/journal.pone.0207706 (PMC6281236; doi:10.1371/journal.pone.0207706)
Supplement: S1 Table — Items translated into Finnish (F) used in the current study are provided in italics. Subscales of each item are provided in parentheses. (PDF) [file pone.0207706.s001.pdf]

S1 Table. Items of the Self-Compassion Scale (Neff, 2003) with Likert scale used in W1 and W2.

Items translated into Finnish (F) used in the current study are provided in italics. Subscales of each item are provided in parentheses.

1. I'm disapproving and judgmental about my own flaws and inadequacies.
   (F) *En hyväksy omia vikojani ja puutteitani vaan tuomitsen ne*. (Self-judgment)

2. When I'm feeling down I tend to obsess and fixate on everything that's wrong.
   (F) *Allapäin ollessani kiinnitän huomioni pakkomielteisesti kaikkeen, mikä on vialla.* (Over-identification)

3. When things are going badly for me, I see the difficulties as part of life that everyone goes through.
   (F) *Kun minulla menee huonosti, ajattelen että vaikeudet ovat osa elämää ja kaikki joutuvat niitä joskus kokemaan*. (Common humanity)

4. When I think about my inadequacies, it tends to make me feel more separate and cut off from the rest of the world.
   (F) *Kun ajattelen vaikeuksiani, alan kokea erillisyyttä muihin ihmisiin.* (Isolation)

5. I try to be loving towards myself when I'm feeling emotional pain.
   (F)*Yritän suhtautua itseeni rakastavasti, kun tunnen henkistä kipua.* (Self-kindness)

6. When I fail at something important to me I become consumed by feelings of inadequacy.
   (F) *Kun epäonnistun jossain minulle tärkeässä asiassa, riittämättömyyden tunne valtaa minut.* (Over-identification)

7. When I'm down and out, I remind myself that there are lots of other people in the world feeling like I am.
   (F) *Kun olen maassa, muistutan itseäni siitä, että maailmassa on paljon ihmisiä, jotka tuntevat samoin.* (Common humanity)

8. When times are really difficult, I tend to be tough on myself.
   (F) *Kun minulla on vaikeaa, olen yleensä ankara itselleni.* (Self-judgment)

9. When something upsets me I try to keep my emotions in balance.
   (F) *Kun jokin järkyttää minua, yritän pitää tunteeni tasapainossa.* (Mindfulness)

10. When I feel inadequate in some way, I try to remind myself that feelings of inadequacy are shared by most people.
    (F) *Kun tunnen itseni riittämättömäksi, yritän muistuttaa itseäni siitä, että suurin osa ihmisistä tuntee riittämättömyyttä.* (Common humanity)

11. I'm intolerant and impatient towards those aspects of my personality I don't like.
    (F) *Suhtaudun suvaitsemattomasti ja kärsimättömästi niihin luonteenpiirteisiini, joista en pidä.* (Self-judgment)

12. When I’m going through a very hard time, I give myself the caring and tenderness I need.
(F) *Kun on todella vaikeaa, suon itselleni tarvitsemaani huolenpitoa ja hellyyttä.* (Self-kindness)

13. When I’m feeling down, I tend to feel like most other people are probably happier than I am.
(F) *Kun olen maassa, minusta tuntuu, että suurin osa muista on todennäköisesti onnellisempia kuin minä.* (Isolation)

14. When something painful happens I try to take a balanced view of the situation.
(F) *Kun jotain tuskallista tapahtuu, pyrin tarkastelemaan tapahtunutta monelta eri kannalta.* (Mindfulness)

15. I try to see my failings as part of the human condition.
(F) *Yritän nähdä epäonnistumiseni osana ihmisyyttä.* (Common humanity)

16. When I see aspects of myself that I don’t like, I get down on myself.
(F) *Kun tunnistan itsessäni piirteitä, joista en pidä, olen ankara itselleni.* (Self-judgment)

17. When I fail at something important to me I try to keep things in perspective.
(F) *Kun epäonnistun jossain itselleni tärkeässä asiassa, pyrin pitämään asiat oikeissa mittasuhteissa.* (Mindfulness)

18. When I’m really struggling, I tend to feel like other people must be having an easier time of it.
(F) *Kun minulla on todella vaikeaa, ajattelen yleensä, että toisilla ihmisillä on varmasti helpompaa.* (Isolation)

19. I’m kind to myself when I’m experiencing suffering.
(F) *Kun kärsin, olen kiltti itselleni.* (Self-kindness)

20. When something upsets me I get carried away with my feelings.
(F) *Kun jokin järkyttää minua, tunteet vievät minut mukanaan.* (Over-identification)

21. I can be a bit cold-hearted towards myself when I'm experiencing suffering.
(F) *Kun kärsin, saatan suhtautua itseeni hieman kylmästi.* (Self-judgment)

22. When I'm feeling down I try to approach my feelings with curiosity and openness.
(F) *Kun mieleni on maassa, yritän tarkastella tunteitani kiinnostuksella ja avoimin mielin.* (Mindfulness)

23. I’m tolerant of my own flaws and inadequacies.
(F) *Suvaitsen omat vikani ja puutteeni.* (Self-kindness)

24. When something painful happens I tend to blow the incident out of proportion.
(F*) Kun jotain tuskallista sattuu, suurentelen tapausta yleensä.* (Over-identification)

25. When I fail at something that's important to me, I tend to feel alone in my failure.
(F) *Kun epäonnistun jossain minulle tärkeässä asiassa, tuntuu, että jään yksin epäonnistumiseni kanssa.* (Isolation)

26. I try to be understanding and patient towards those aspects of my personality I don't like.
(F) *Yritän suhtautua ymmärtäväisesti ja maltillisesti niihin ominaisuuksiini, joista en pidä. (Self-kindness)*

Finnish translation of the Likert scale used in W1 (From 1 ("Hardly ever") to 5 ("Almost always")

(F) 1 = En melkein koskaan, 2 = Hyvin harvoin, 3 = En usein enkä harvoin, 4 = Hyvin usein, 5 = Lähes aina

Finnish translation of the Likert scale used in W2 (From 1 ("Almost never") to 5 ("Almost always")

(F) 1 = Hyvin harvoin, 2 = Toisinaan, 3 = Noin puolet ajasta, 4 = Melko usein, 5 = Lähes aina

---
