# Supplementary material for: Two is more valid than one, but is six even better? The factor structure of the Self-Compassion Scale (SCS)
Source: PLoS One. 2018 Dec 5;13(12):e0207706. doi: 10.1371/journal.pone.0207706 (PMC6281236; doi:10.1371/journal.pone.0207706)
Supplement: S2 Table — (PDF) [file pone.0207706.s002.pdf]

S2 Table. Fully standardized Factor loadings, standard errors and residual variances in the preliminary two-factor model (W1).

Item	Factor loading		SE	Residual variance
	Self-Compassion	Self-Coldness		
SCOMP5	.651		.026	.576
SCOMP12	.639		.024	.591
SCOMP19	.707		.029	.631
SCOMP23	.594		.028	.648
SCOMP26	.656		.024	.570
SCOMP1		.678	.021	.540
SCOMP8		.739	.019	.454
SCOMP11		.593	.025	.648
SCOMP16		.710	.022	.496
SCOMP21		.733	.018	.463
SCOMP3	.536		.029	.712
SCOMP7	.452		.033	.796
SCOMP10	.526		.030	.723
SCOMP15	.707		.022	.501
SCOMP4		.643	.021	.586
SCOMP13		.656	.023	.569
SCOMP18		.618	.026	.618
SCOMP25		.676	.020	.542
SCOMP9	.448		.036	.799
SCOMP14	.619		.027	.617
SCOMP17	.614		.029	.623
SCOMP22	.612		.026	.625
SCOMP2		.730	.018	.467
SCOMP6		.681	.022	.536
SCOMP20		.568	.029	.678
SCOMP24		.486	.030	.764
