# Supplementary material for: Two is more valid than one, but is six even better? The factor structure of the Self-Compassion Scale (SCS)
Source: PLoS One. 2018 Dec 5;13(12):e0207706. doi: 10.1371/journal.pone.0207706 (PMC6281236; doi:10.1371/journal.pone.0207706)
Supplement: S3 Table — (PDF) [file pone.0207706.s003.pdf]

S3 Table. Fully standardized Factor loadings, standard errors and residual variances in the two-factor model with seven residual correlations (between item 7 and 10, items 26 and 23, items 9 and 17 (loading on self-compassion factor) and items 16 and 8, items 18 and 13 items 1 and 2, and finally, items 4 and 6 (loading on self-coldness factor) (W1).

	Factor loading			
	Self-Compassion	Self-Coldness	SE	Residual variance
SCOMP5	.658		.026	.576
SCOMP12	.644		.024	.585
SCOMP19	.619		.028	.617
SCOMP23	.577		.028	.668
SCOMP26	.648		.024	.580
SCOMP1		.641	.022	.589
SCOMP8		.711	.022	.495
SCOMP11		.597	.026	.643
SCOMP16		.682	.024	.535
SCOMP21		.743	.018	.448
SCOMP3	.528		.030	.721
SCOMP7	.443		.031	.804
SCOMP10	.482		.030	.768
SCOMP15	.709		.023	.497
SCOMP4		.625	.022	.609
SCOMP13		.654	.023	.573
SCOMP18		.614	.024	.623
SCOMP25		.691	.020	.522
SCOMP9	.424		.032	.820
SCOMP14	.625		.033	.609
SCOMP17	.598		.030	.642
SCOMP22	.618		.026	.618
SCOMP2		.700	.026	.509
SCOMP6		.679	.029	.539
SCOMP20		.587	.027	.656
SCOMP24		.498	.027	.752
