# Supplementary material for: Two is more valid than one, but is six even better? The factor structure of the Self-Compassion Scale (SCS)
Source: PLoS One. 2018 Dec 5;13(12):e0207706. doi: 10.1371/journal.pone.0207706 (PMC6281236; doi:10.1371/journal.pone.0207706)
Supplement: S4 Table — (PDF) [file pone.0207706.s004.pdf]

S4 Table. Fully standardized Factor loadings, standard errors and residual variances in the preliminary two-factor model (W2).

Item	Factor loading		SE	Residual variance
	Self-Compassion	Self-Coldness		
SCOMP5	.701		.022	.509
SCOMP12	.687		.023	.528
SCOMP19	.607		.032	.632
SCOMP23	.655		.026	.571
SCOMP26	.694		.026	.518
SCOMP1		.721	.021	.480
SCOMP8		.754	.019	.431
SCOMP11		.626	.028	.608
SCOMP16		.761	.018	.421
SCOMP21		.710	.023	.495
SCOMP3	.607		.031	.632
SCOMP7	.561		.029	.686
SCOMP10	.598		.027	.643
SCOMP15	.766		.019	.413
SCOMP4		.705	.021	.503
SCOMP13		.702	.023	.507
SCOMP18		.637	.026	.594
SCOMP25		.706	.020	.501
SCOMP9	.564		.036	.682
SCOMP14	.665		.025	.558
SCOMP17	.698		.027	.512
SCOMP22	.682		.023	.534
SCOMP2		.781	.017	.390
SCOMP6		.756	.019	.428
SCOMP20		.587	.029	.656
SCOMP24		.544	.028	.704
