# Supplementary material for: Two is more valid than one, but is six even better? The factor structure of the Self-Compassion Scale (SCS)
Source: PLoS One. 2018 Dec 5;13(12):e0207706. doi: 10.1371/journal.pone.0207706 (PMC6281236; doi:10.1371/journal.pone.0207706)
Supplement: S5 Table — (PDF) [file pone.0207706.s005.pdf]

S5 Table. Fully standardized Factor loadings, standard errors and residual variances in the two-factor model with seven residual correlations (between item 7 and 10, items 26 and 23, items 9 and 17 (loading on self-compassion factor) and items 16 and 8, items 18 and 13 items 1 and 2, and finally, items 4 and 6 (loading on self-coldness factor) (W2).

Item	Factor loading		SE	Residual variance
	Self-Compassion	Self-Coldness		
SCOMP5	.695		.022	.517
SCOMP12	.682		.024	.535
SCOMP19	.655		.030	.571
SCOMP23	.636		.025	.596
SCOMP26	.681		.026	.537
SCOMP1		.692	.022	.522
SCOMP8		.728	.021	.470
SCOMP11		.620	.028	.615
SCOMP16		.735	.021	.460
SCOMP21		.713	.024	.492
SCOMP3	.629		.031	.604
SCOMP7	.554		.027	.694
SCOMP10	.548		.028	.700
SCOMP15	.761		.019	.421
SCOMP4		.713	.020	.492
SCOMP13		.700	.020	.511
SCOMP18		.626	.027	.608
SCOMP25		.730	.021	.481
SCOMP9	.569		.033	.676
SCOMP14	.686		.024	.529
SCOMP17	.701		.027	.508
SCOMP22	.686		.022	.530
SCOMP2		.763	.019	.418
SCOMP6		.766	.019	.414
SCOMP20		.598	.030	.643
SCOMP24		.554	.028	.694
